# Determination of the glycoprotein specificity of lectins on cell membranes through oxidative proteomics†

**DOI:** 10.1039/d0sc04199h

**Published:** 2020-08-17

**Authors:** Yixuan Xie, Ying Sheng, Qiongyu Li, Seunghye Ju, Joe Reyes, Carlito B. Lebrilla

**Affiliations:** Department of Chemistry, University of California Davis Davis California USA cblebrilla@ucdavis.edu; Department of Chemistry, Biochemistry, Molecular, Cellular and Developmental Biology Graduate Group, University of California Davis Davis California USA; Department of Biochemistry, University of California Davis Davis California USA; Marine Science Institute, University of the Philippines Diliman Quezon City Philippines

## Abstract

The cell membrane is composed of a network of glycoconjugates including glycoproteins and glycolipids that presents a dense matrix of carbohydrates playing critical roles in many biological processes. Lectin-based technology has been widely used to characterize glycoconjugates in tissues and cell lines. However, their specificity toward their putative glycan ligand and sensitivity *in situ* have been technologically difficult to study. Additionally, because they recognize primarily glycans, the underlying glycoprotein targets are generally not known. In this study, we employed lectin proximity oxidative labeling (Lectin PROXL) to identify cell surface glycoproteins that contain glycans that are recognized by lectins. Commonly used lectins were modified with a probe to produce hydroxide radicals in the proximity of the labeled lectins. The underlying polypeptides of the glycoproteins recognized by the lectins are oxidized and identified by the standard proteomic workflow. As a result, approximately 70% of identified glycoproteins were oxidized *in situ* by all the lectin probes, while only 5% of the total proteins were oxidized. The correlation between the glycosites and oxidation sites demonstrated the effectiveness of the lectin probes. The specificity and sensitivity of each lectin were determined using site-specific glycan information obtained through glycomic and glycoproteomic analyses. Notably, the sialic acid-binding lectins and the fucose-binding lectins had higher specificity and sensitivity compared to other lectins, while those that were specific to high mannose glycans have poor sensitivity and specificity. This method offers an unprecedented view of the interactions of lectins with specific glycoproteins as well as protein networks that are mediated by specific glycan types on cell membranes.

## Abstract 2

A lectin proximity oxidative labeling (Lectin PROXL) tool was developed to identify cell surface glycoproteins that contain glycans that are recognized by lectins.

## Graphical abstract

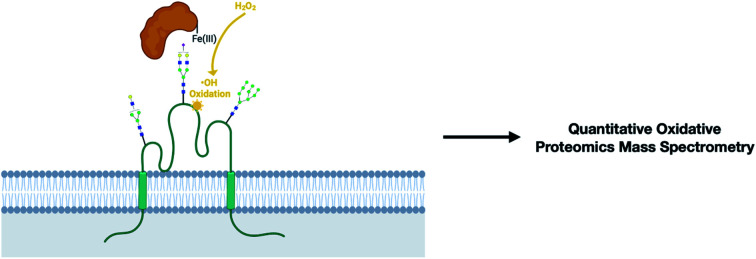

## Introduction

The carbohydrate layer on the cell surface that is anchored by protein and lipid scaffolds is involved in a host of important and central cellular processes, including cellular adhesion, immune defense, and cell permeability.^1–3^ Extensive covalent and non-covalent interactions occur on the cell membrane that defines the topology and availability of glycan-binding sites and antigens. Cell-binding proteins such as cadherins and integrins are glycosylated with glycans centrally involved in their function.^4,5^ Enzymes used for protection such as DPP IV (dipeptidyl peptidase IV) and IAP (alkaline phosphatase) are also glycosylated with their function affected by glycosylation.^6^

Lectin-based techniques, including lectin microarray, lectin-affinity enrichment, and enzyme-linked lectin assay (ELLA), are extensively used for studying specific glycan structures *in vitro*.^7^ However, due to the transient and weak nature of glycan-mediated interactions, glycans need to be in their native environment to resolve the structure and dynamics of such interaction. Various glycoprotein models have also been introduced to mimic the spatial distribution of glycan epitopes on natural glycoconjugate ligands.^8–10^ Electrochemical imaging has been widely employed to visualize and characterize glycoconjugates *in situ*.^11^ Recently, Han *et al.* developed a method involving using laser cleavable lectin probes for glycan detection at the single cell level through mass spectrometry (MS)-based analysis.^12^ These methods rely highly on the putative specificities of lectins towards glycoconjugates, however the *in situ* targets of the lectins are generally unknown thereby hindering the broader applications of these methods.

Several strategies have been developed to correlate the visual information with detailed protein information. In particular, metabolic labeling of bioorthogonal reporters has been introduced to study the *in situ* interactions between lectins and glycans. Paulson and co-workers applied photocrosslinking sialic acids to identify the *cis*- and *trans*-targets of Siglec-2 *in situ*.^13,14^ Kohler and co-workers created complementary methods with photocrosslinking sugars modified at the C-5 position.^15^ However, these investigations were focused on specific lectin-glycoprotein partners.

Covalent labeling has been used more broadly to obtain interactive relationships but in a more generalized manner by providing proximity information of proteins involved.^16^ In particular, oxidative labeling using hydroxyl radicals has been extensively used to examine protein–protein interactions.^17^ Fenton reactions with iron, cobalt, chromium, nickel, copper, and manganese have been used for the production of hydroxyl radicals upon reaction with hydrogen peroxide.^18–21^ Due to the higher tolerance of iron under physiological conditions, it has been more commonly used as a catalyst. For example, Zhu *et al.* characterized the porin OmpF protein structure *in situ* through the reaction of hydroxyl radicals generated by hydrogen peroxide and iron.^22^

**Fig. 1:**
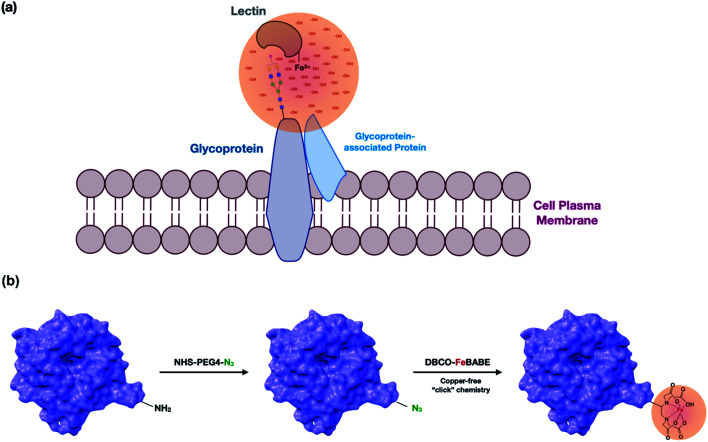
(a) A representation of the lectin probe attached to a target glycan and oxidizing the underlying polypeptide scaffold. The Fe(iii)-modified lectin probes were the cell supernatant and allowed to interact with the glycan target. Hydroxyl radicals were produced at the metal site creating a concentration of radicals that oxidize the proteins in proximity. The membrane proteins are isolated and analyzed by LC-MS. Oxidized proteins are identified and quantified. (b) The modification involved the introduction of the azido group into a primary amine on lysine followed by conjugation of DBCO-FeBABE to the lectin *via* copper-free “click” chemistry.

In this research, we used oxidative labeling by reacting a lectin with dibenzocyclooctyne-FeBABE (DBCO-FeBABE) as a probe to generate hydroxyl radicals (Fig. 1a). A similar reagent, iron (S)-1-(*p*-bromoacetamidobenzyl) EDTA (FeBABE), was synthesized previously and coupled to proteins to identify protein-associated interactions.^23^ The primary amine on the lectin was functionalized with azide, followed by conjugation to the azido group to form oxidative probes on the modified lectins (Fig. 1b). The probe produced hydroxyl radicals when hydrogen peroxide was introduced. Thus, proteins near the lectins were oxidized by the generated hydroxyl radicals and the oxidatively modified side chains were characterized using nanoLC-MS. The lectin proximity oxidative labeling (Lectin PROXL) method extends a previous one mapping the potential sialic acid-associating protein on the cell surface.^24^ Eight commonly used lectins were chosen to identify the specific glycoprotein targets of each lectin (Table 1). *Sambucus nigra* agglutinin (SNA) binds to sialic acid with the α(2,6) linkage preference, and *Maackia amurensis* leukoagglutinin (MAL) binds to α(2,3) sialic acid.^25,26^*Aleuria aurantia* lectin (AAL) binds to fucose in general, while *Pisum sativum* agglutinin (PSA) prefers core fucosylation.^27^ Both *Phaseolus vulgaris* leucoagglutinin (PHA-L) and *Phaseolus vulgaris* erythroagglutinin (PHA-E) bind to galactose, but PHA-L prefers tri/tetra-antennary *N*-glycans and PHA-E has a higher affinity toward bi-antennary *N*-glycan.^28,29^*Hippeastrum hybrid* lectin (HHL) binds to high mannose type *N*-glycans through recognizing α(1,3) and α(1,6) mannose.^30^*Wheat germ* agglutinin (WGA) with *N*-acetylglucosamine and sialic acid binding properties was chosen to investigate the general targets.^31^ Lectin PROXL was applied to evaluate the interactions between lectins and glycoproteins to provide the protein targets on the cell surface, and the functional analysis of specifically oxidized proteins provided networks that were mediated by glycans on cell membranes.

**Table 1:** The lectins used in the study and their putative glycan targets

Lectin	Origin	Putative monosaccharide target(s)	Putative *N*-glycan targets
WGA	*Wheat germ*	*N*-acetylglucosamine and sialic acid	N-glycans in general
SNA	*Sambucus nigra*	Sialic acid	α(2,6) sialylated glycans
MAL	*Maackia amurensis*	Sialic acid	α(2,3) sialylated glycans
AAL	*Aleuria aurantia*	Fucose	Fucosylated glycans in general
PSA	*Pisum sativum*	Fucose	α(1,6) fucosylated glycans
HHL	*Hippeastrum hybrid*	Mannose	High mannose glycans
PHA-E	*Phaseolus vulgaris*	Galactose	Biantennary complex-type and bisecting glycans
PHA-L	*Phaseolus vulgaris*	Galactose	Tri/tetra-antennary complex-type glycans

## Results

### Production of lectin PROXL probes

To confirm the efficacy of the overall reaction, bovine serum albumin (BSA) was used as a model protein and characterized by nanoLC-MS. The modification of the protein with azide-PEG4-NHS was performed and the protein was subjected to conjugation with the probe. As shown in Fig. S1,† the tandem MS/MS data confirmed the presence of the azido group modification on BSA (+273.13 Da), as well as the conjugation of DBCO (+549.26 Da) on the K437 residue. A greater than 80% conversion was achieved for this site as determined by the intensities of modified over wild-type peptides. Additionally, there were two other sites that reacted and yielded an incorporation of 60 to 70%. Missed cleavages were observed at the modified lysine residues.

To determine whether the binding properties of lectins were affected by the modifications, we prepared DBCO-cy3-labeled SNA using DBCO-cy3 and applied it towards PNT2 cells. SNA has specificities towards α(2,6) sialic acids. Cells were fixed with formaldehyde, treated with the lectin, and then stained with Hoechst to observe the nucleus. No significant decrease in the fluorescence intensity was observed in the modified lectin thereby indicating that the modification did not alter the interactions between the lectin and its receptors on the cell surface (Fig. S2a and b†), although it is duly noted that the method does not yield accurate quantitation so that some modulation of the binding may still occur.

### Validation of the lectin PROXL method

The condition for oxidative labeling was optimized by varying the hydrogen peroxide concentrations and the reaction times, which govern the flux and diffusion distances of the hydroxyl radicals. Higher concentrations of hydrogen peroxide yielded longer distances for the radicals to travel, while low concentrations yielded shorter distances. Previous studies on the effects of concentrations showed that the radicals can travel as far as 60 Å.^32^ We have previously found that 50–300 μM hydrogen peroxide concentrations and 30 minutes reaction time produce hydroxyl radicals within 20 Å of the probe, which was optimal for identifying proteins that were in the vicinity of the probe.^24^ With the reaction time fixed to 30 minutes, we varied the hydrogen peroxide concentrations from 50 μM to 300 μM. We used WGA for optimization because WGA binds putatively to nearly all glycans through its specificity for *N*-acetylglucosamines (GlcNAc) and sialic acids.

The results indicated that the number of oxidized proteins increased with higher hydrogen peroxide concentrations but did not increase significantly more at concentrations greater than 100 μM. To monitor the extent of the reactions, we determined the increase in the number of oxidation sites as well as the extent of oxidations on specific sites (Fig. S3a†). The subcellular locations of oxidized proteins were annotated using STRING software, and we found that oxidations on the membrane proteins were predicted to be primarily occurring on the extracellular sides of the proteins. We also measured the distances between oxidation sites and glycosites for proteins targeted by the WGA probe with 3D protein models using ChimeraX.^33^ Consequently, most of the oxidation sites were within 25 Å of the glycosylation sites, and it showed that the optimum distance was 20 Å after applying the Gaussian fitting curve, which confirms our previously published results (Fig. S4a†). Based on these results, we chose 100 μM and 30 minutes as the optimal reaction conditions for Lectin PROXL. The results also suggested that nonspecific oxidation can be monitored and overoxidation can be avoided by limiting the reaction time and the hydrogen peroxide concentration. To confirm the reproducibility of the identification, WGA probe oxidized protein samples were analyzed in triplicate. As shown in the heatmap in Fig. S3b,† the extent of oxidation on specific sites with multiple proteins did not vary significantly thereby demonstrating the repeatability of the analysis.

It was noticed that methionine was the most commonly oxidized amino acid followed by others including phenylalanine and tyrosine, which were oxidized to lower degrees (Fig. S4b†). The extent of oxidation among these amino acids with methionine being the most oxidized is consistent with a number of other similar experiments using hydroxyl radical reagents.^16^ For example in a previous study on Calmodulin-protein binding, methionine residues were indeed the most oxidized by hydroxyl radicals followed by phenylalanine and tyrosine.^34^

### Oxidative labeling of glycoproteins on the cell surfaces of PNT2 cell lines

**Fig. 2:**
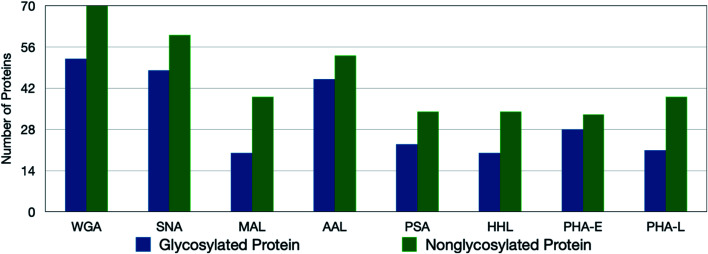
The number of oxidized glycoproteins and nonglycosylated proteins in PNT2 cell lines using lectin probes. Although more nonglycosylated proteins are oxidized, the degree of oxidation on the target glycoproteins is significantly greater.

To monitor the overall oxidation of the proteins, quantitative standard proteomic analysis was performed. For PNT2 cells, the experiments typically yielded more than 200 oxidized proteins for all eight lectins (ESI 1–2†). Approximately 70% of identified glycoproteins were oxidized by the lectin probe, while only 5% of the nonglycosylated proteins were oxidized (Fig. 2). The glycosylated proteins contained the target glycans, and these oxidized glycoproteins were quantified using Byologic software (Fig. S5†). On the other hand, the oxidized nonglycosylated proteins were likely those that interacted with the target glycoproteins. Background oxidation was identified and noted through the use of control experiments.

We further observed a general correlation between the number of glycosylation sites and the extent of oxidation on the glycoprotein. Of all glycoproteins containing only one glycosylation site around 35% of the proteins showed oxidation, while for those containing more than one glycosite over 75% of these glycoproteins were oxidized. For example, CD166 (CD166 antigen) with *N*-glycans at N167, N265, N361, and N480 when reacted with the SNA probe yielded four oxidation sites. Conversely, the protein TSN13 (tetraspanin-13) with sialofucosylated glycans at a single site N137 was not oxidized by SNA or any of the other lectins.

### Determination of the relationship between the sites of glycosylation and the sites of oxidation

To obtain the site-specific information of oxidized glycoproteins, including peptide sequences, glycosites, and glycan compositions, we employed a secondary glycoproteomic analysis of the cell membrane proteins (shown in ESI 3†). The membrane proteins were digested, and the glycopeptides were enriched through hydrophilic interaction chromatography (HILIC) using solid-phase extraction (SPE). The enriched fractions were analyzed using nanoLC-MS and the sites of oxidation were examined relative to the sites of glycosylation to determine the spatial relationship between them. For example, SNA is a lectin known to recognize sialylated glycans. Sialylated glycans including Man_(3)_Gal_(2)_GlcNAc_(4)_Sia_(1)_, Man_(3)_Gal_(2)_GlcNAc_(4)_Sia_(2)_, and Man_(3)_Gal_(3)_GlcNAc_(5)_Sia_(3)_, were found specifically at N343 on ITA2 (integrin alpha-2), while high-mannose type glycans Man_(8)_GlcNAc_(2)_ and Man_(7)_GlcNAc_(2)_ were found at N432 (Fig. S4c†). From the oxidation results, we found that ITA2 was oxidized by SNA lectin at T337, which was closer to the site of sialylation than mannosylation.

**Fig. 3:**
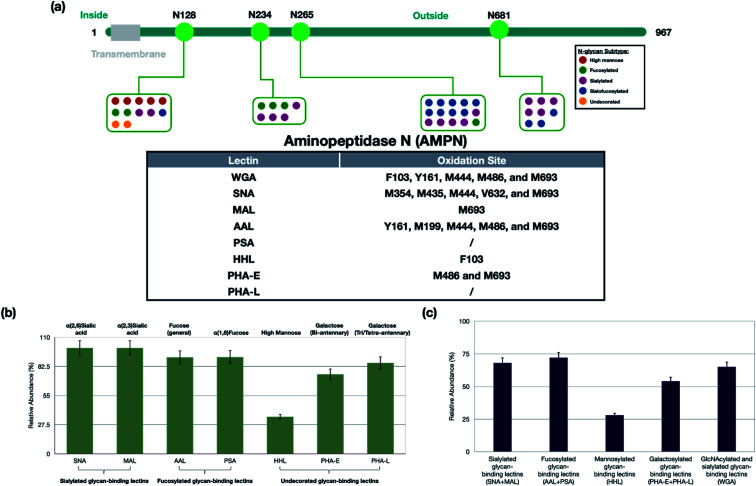
(a) The relationship between the sites of glycosylation and the sites of oxidation on an example protein, AMPN. The oxidation site on the glycoprotein was highly dependent on the distribution of different types of glycans. (b) The glycoprotein specificity of lectins on PNT2 cells. The specificity of the lectins was determined as the number of oxidized glycoproteins containing the putative glycan structure *versus* all oxidized glycoproteins. Most of the lectins showed high specificity towards the putative target glycoproteins (>70%). The lectin HHL was the exception, which recognized only 30% high mannose glycans. The error bars were obtained based on triplicate results. (c) The sensitivity of each lectin was determined by the number of oxidized glycoproteins containing the putative target *versus* the total number of glycoproteins (oxidized and not oxidized) containing the putative target. The lectins were grouped because linkages of, for example, the fucose were not known. The sensitivity is highest for AAL and PSA (fucose binding lectins) and lowest for HHL (mannose binding lectins).

AMPN (aminopeptidase N) is a highly glycosylated protein with *N*-glycans distributed over four glycosites including N128, N234, N265, and N681 (Fig. 3a). The SNA probe oxidized the protein extensively at M354, M435, M444, V632, and M693, which were all near the glycosites associated with sialylated glycans. Another sialic acid recognizing lectin, MAL with the putative specificity for α(2,3) sialic acid labeled the protein only at M693. These results suggested that the sialic acids on N265 are likely α(2,6) sialic acid due to the proximity, while the sialylated glycans at N681 likely contained both α(2,6) and α(2,3) sialic acids due to the oxidation at M693 for both probes. High mannose *N*-glycans were also detected at N128 exclusively. Indeed, the high mannose recognizing lectin HHL yielded oxidation of AMPN at F103. Glycoproteomic analysis also revealed fucosylated glycans at the same four glycosites. Similarly, the oxidation results were consistent with the localization by the fucose-binding lectin AAL at Y161, M199, M444, M486, and M693. Interestingly, the galactose-binding lectin, PHA-E, oxidized the AMPN protein only at M486 and M693, although bi-antennary glycans were observed at all four glycosites. The WGA probe was expected to oxidize the glycoprotein at the same sites as all the other lectins. WGA is generally believed to bind to all *N*-glycans through its affinity with the GlcNAc and sialic acids. As a result, the WGA lectin probe was found to oxidize AMPN protein at F103, Y161 and M444, M496, and M693 consistent with expectations.

Not all lectins were equally effective in binding to their putative targets. The lectin PSA is specific towards core fucosylation, however despite the large number of fucosylated glycans AMPN was not oxidized by PSA. The lack of reactivity could be attributed to two factors. One is that these glycans contained no core fucosylation. Or, the core fucose was deep within the fold of the protein and could not be accessed by the lectin. As core fucosylation is much more common than antenna fucosylation, we believed that the latter was more correct. Below, we show modeling calculations that exhibit this behavior. Another lectin, PHA-L, with a specificity towards galactose on termini of tri- and tetra-antennary did not yield oxidized AMPN products, despite the presence of tri- and tetra-antennary structures. In these glycans, the termini contained fucose and sialic acids, and the presence of these residues likely block the terminal galactose from binding with the lectin. It has been shown that the affinity of this lectin toward galactose is diminished by the presence of the sialic acid and fucose on the galactose.^35^

### Glycoprotein specificity of lectins

For the specificity of the lectins, we determined the number of oxidized glycoproteins containing the putative glycan structure *versus* all oxidized glycoproteins. For example, all 48 glycoproteins that were oxidized by SNA contained sialylated glycans (100% specificity), while only 35% (7 out of 20) of glycoproteins oxidized by HHL contained high-mannose type glycans (Fig. 3B). For clarity, we grouped the lectins into four types, namely sialylated-binding lectins (SNA and MAL), fucosylated-binding lectins (AAL and PSA), undecorated glycan-binding lectins (PHA-E, PHA-L, and HHL) and a glycan-binding lectin with broad specificities (WGA).

The lectins SNA and MAL both bind glycans with sialic acids, however SNA is specific for α(2,6) while MAL is specific for α(2,3). From the proteomic analysis, all glycoproteins oxidized by SNA were sialylated as were all 20 by MAL. The larger number of glycoproteins marked by SNA compared to MAL implied the greater presence of α(2,6) *versus* α(2,3) in PNT2 cells. These results were further validated for this cell line with fluorescence labeling, which showed greater fluorescence with SNA compared to MAL (Fig. S2c†). Of the glycoproteins oxidized by MAL, 17 were also oxidized by SNA suggesting the presence of both linkages in those proteins (Fig. S6a†). The three glycoproteins uniquely oxidized by MAL suggested that these sialylated glycoproteins contained primarily α(2,3) sialic acid. To confirm this notion, we treated the glycoprotein from PNT2 cells with α(2,3) sialidase.^36^ As shown in ESI 4,† one of the proteins, MPRD (protein cation-dependent mannose-6-phosphate receptor) was found to have a sialylated glycan at N83 corresponding to Hex_(5)_HexNAc_(4)_Fuc_(1)_NeuAc_(1)._ Treatment of the glycopeptide mixture with the sialidase resulted in the loss of the sialic acid and the appearance of the desialylated glycopeptide confirming the linkage of this glycoform. Other glycopeptides belonging to the three proteins yielded similar results.

**Fig. 4:**
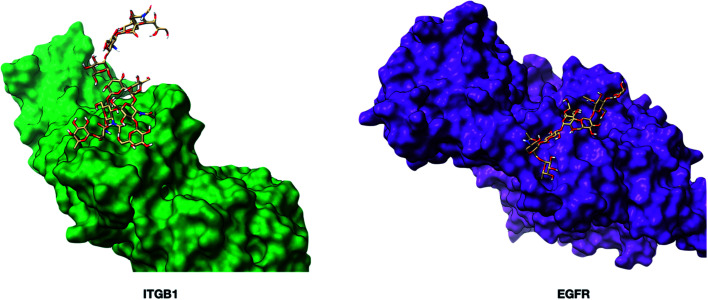
The three-dimensional structures of glycoproteins ITGB1 (integrin beta-1, PDB: 3VI3) and EGFR (epidermal growth factor receptor, PDB: 1NQL) containing the glycan Man_(3)_Gal_(2)_GlcNAc_(4)_Fuc_(1)_Sia_(1)_. The glycoprotein models were built using Glycam (http://glycam.org). The core fucose on EGFR was predicted to be non-accessible to the PSA lectin.

The fucose-binding lectins, AAL and PSA, yielded 45 and 23 oxidized proteins, respectively. AAL has broad specificities towards the core and antenna fucose, while PSA prefers mainly core fucose. The 21 glycoproteins marked by PSA were found in AAL proteins (Fig. S6b†). The oxidized glycoproteins observed in common were found to have monofucosylated glycans, while the glycoproteins that were uniquely labeled by AAL were dominated by difucosylated and trifucosylated structures that likely contain fucose at the antenna. Not all glycoproteins with the monofucosylated glycans were labeled by PSA. For example, protein ITGB1 (integrin beta-1) with Hex_(5)_HexNAc_(4)_Fuc_(1)_Sia_(2)_ at N97 was oxidized at L108, while EGFR (epidermal growth factor receptor) with the same glycan composition at N528 did not yield oxidized peptides. However, EGFR was multiply fucosylated and was oxidized by AAL. There are at least two reasons for why EGFR was not oxidized by PSA: the lone fucose was not at the core, or the core fucose was not accessible due to steric shielding. To investigate the accessibility of the core fucose on these two proteins, we built two glycoprotein models and minimized the energy using Glycam.^37^ As shown in Fig. 4, fucose on EGFR was predicted to be sterically obscured by K538 and L541 side chains in the vicinity of the *N*-glycan site. Conversely, the core fucose on ITGB1 was unhindered and readily accessible. The results suggest that protein folding and spatial accessibility of glycans could be factors that hinder recognition by lectins.

Galactosylated structures are putatively recognized by lectins PHA-L and PHA-E, although the two lectins have different affinities towards various numbers of antennas. PHA-L favors tri/tetra-antennary, while PHA-E binds with bi-antennary structures. We found that glycoproteins uniquely oxidized by PHA-E indeed contained mainly bi-antennary *N*-glycans, while tri- and tetra-antennary *N*-glycans were the primary targets of the PHA-L probe (figure not shown). In general, PHA-E yielded more oxidized glycoproteins, which suggested that the cell line had more bi-antennary instead of tri/tetra-antennary structures. We further employed *N*-glycomic analysis on PNT2, and we found that the cells indeed had more bi-antennary *N*-glycans such as Man_(3)_Gal_(2)_GlcNAc_(4)_Fuc_(1)_ and Man_(3)_Gal_(2)_GlcNAc_(4)_Fuc_(1)_Sia_(1)_ than higher antennary structures (Fig. S7†).

Nearly all the lectins had very high specificity towards the putative protein-associated glycan with perhaps one exception, namely HHL. HHL is commonly used to identify high mannose type *N*-glycans, because it can recognize both (α1,3) and (α1,6) mannose structures. Due to the lack of high-mannose type glycans on the PNT2 cell surface, HHL only generated 20 oxidized glycoproteins. Within this group, only seven glycoproteins were found to have high-mannose structures. This apparent lack of specificity suggests that HHL binding is perhaps not limited to high-mannose type glycans, and it has been shown that HHL can also bind to *N*-glycans with terminal galactose and sialic acid.^38^ Therefore, caution should be taken when HHL is used strictly for monitoring the amount of high mannose structures.

WGA is a lectin with broad specificities and is widely used as a probe for monitoring all *N*-glycans. Probing PNT2 with WGA yielded over 50 oxidized glycoproteins. Indeed, WGA yielded the highest number of oxidized glycoproteins among all eight lectins investigated. It should be further noted that the glycoproteins oxidized by the WGA probe were also oxidized with other lectin probes thereby confirming the broad specificity of WGA towards *N*-glycans. However, not all glycoproteins oxidized by the other lectins were oxidized by the WGA probe. To determine how WGA differentiates glycoproteins, we employed glycoproteomic analysis of the cell line. We found that the glycoproteins not oxidized by WGA contained primarily complex type *N*-glycans. For example, the complex type *N*-glycan, Hex_(5)_HexNAc_(4)_Fuc_(2)_NeuAc_(1)_, was found on N166 of ECE1 (endothelin-converting enzyme 1). While the parent protein was oxidized by AAL, it was not marked by WGA. On the other hand, most of the WGA oxidized glycoproteins contained hybrid-type structures. For example, the protein EPCAM (epithelial cell adhesion molecule), which was oxidized by WGA, contained hybrid *N*-glycans, Man_(4)_Gal_(1)_GlcNAc_(3)_Sia_(1)_ and Man_(4)_Gal_(1)_GlcNAc_(3)_Fuc_(1)_, on N111. Although WGA is commonly used to monitor generally *N*-glycosylation, our results suggested that WGA prefers the hybrid type *N*-glycans over the complex type *N*-glycans and supported the previous studies conducted by Hirabayashi and co-workers.^39^

We further obtained the sensitivity of Lectin PROXL by determining the fraction of glycoproteins containing the putative target that were oxidized *versus* the total (oxidized and non-oxidized) of the same glycoprotein groups. The fraction of glycoproteins oxidized by the probe revealed the general sensitivity of the lectin, which was generally determined to be in the range of 60–70% (Fig. 3c). For WGA, the sensitivity was 65% signifying the fraction of the glycoproteins that were oxidized by the probe. Thus, its utility as a general *N*-glycan lectin is moderate at least for this cell line. The sensitivity of both SNA and MAL was higher at 68%. Interestingly, the fucose binding lectins, AAL and PSA, were found to be even higher at approximately 72%. The mannose binding protein HHL had the lowest sensitivity at 28% which was due to the low expression of high mannose on PNT2 cells.

### Glycoprotein-protein interactions on cell membranes are probed by lectins

A small fraction of the nonglycosylated proteins oxidized by the probes were found to be primarily glycan binding proteins that were oxidized due to their proximity to the glycoproteins. For example, ANXA 2 (Annexin II) is a protein with cationic binding function and is oxidized by SNA. Annexins are a group of calcium-dependent membrane proteins that associate with other proteins. They have been shown to have glycan-binding properties with affinities toward negatively charged glycans such as sialylated glycans and heparan.^40,41^

SNA and MAL had an 85% overlap in the oxidized glycoprotein targets. The nonglycosylated proteins had a similarly large overlap (over 70%) (Fig. S8a†). We compared the nonglycosylated proteins oxidized by SNA and MAL to those previously identified as potentially sialic acid binding proteins using an orthogonal approach. In an earlier study, sialic acids were linked to an Fe^3+^ probe to mark (oxidize) proteins that were in the proximity of sialic acids.^24^ By comparing the current results with the previous results, we found more than 60% overlap in the proteins identified further supporting the notion that oxidized nonglycosylated proteins are those that interact with the primary targeted glycoproteins (Fig. S9†).

The nonglycosylated proteins oxidized by fucose-recognizing lectins (AAL and PSA) were similarly believed to be fucose-binding proteins. The overlap in the nonglycosylated proteins between AAL and PSA was over 90% (Fig. S8b†). There was a similarly large overlap (>80%) between fucose-associated and sialic acid-associated proteins (Fig. S10†). The similarities were consistent with the glycosylation in PNT2, of which the majority of glycans are both sialylated and fucosylated, and the large overlap in the oxidized proteins between the two types of lectins was consistent with the specificities of these interactions.

**Fig. 5:**
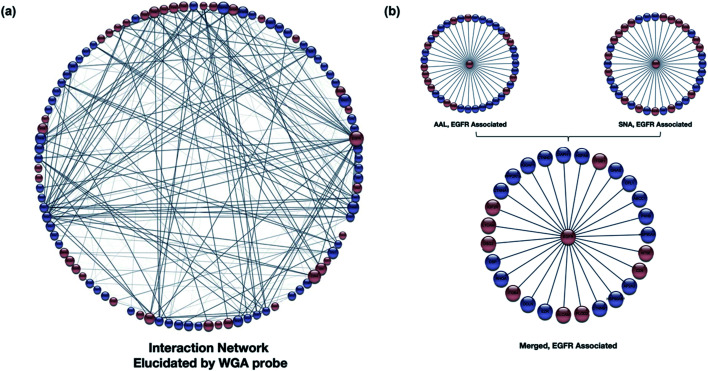
(a) The WGA interaction network was produced using STRING (https://string-db.org).^54^ The software assigns interaction lines when known interactions (literature) are present. The glycoproteins (red) and nonglycosylated proteins (blue) are shown with their respective interactions with the size of the node proportional to the number of interactions. The weight of each protein connection showed the confidence in the interactions. (b) The interaction network of an example protein, EGFR-associated proteins as revealed by AAL and SNA. Over 80% overlap was observed in the two interaction networks illustrating the validity of the method.

Other relationships between the target glycoproteins and the associated (nonglycosylated) proteins were further examined using the STRING software. A general map using WGA with Cytoscape is shown (Fig. 5a), with the glycoproteins in red and nonglycosylated proteins in blue.^42^ The interaction map showed that the proteins (glycosylated and non) were highly interactive and mediated by specific types of glycosylation. Similarly, the interaction maps can be generated using other lectins, such as SNA and AAL (Fig. S11†). More than 75% overlap was observed by comparing the SNA and AAL interaction network (Fig. S12a†), which is consistent with the dominant presence of sialofucosylated glycans. For example, a highly sialofucosylated protein EGFR was found to interact extensively with other nonglycosylated proteins and glycoproteins (Fig. 5b). In contrast, there was less than 20% overlap between high mannose and sialic acid binding lectins (Fig. S12b†). The results confirmed the consistency of Lectin PROXL and suggested that protein–protein interactions can be probed using various lectins and assigned to the mediating glycan type based on the lectin. These results further suggested that the glycan structure could act as the determinant to control the interactions between glycoproteins.

### Application of the method towards the LNCaP cell line

The lectin probes were also applied to the human prostate cancer cell line, LNCaP, to investigate the behavior of lectins with a cell line having different glycocalyx profiles (ESI 5–7†). The LNCaP cell line was found to be highly fucosylated but differed from PNT2 in that it was low in sialylation (Fig. S7†). A smaller subset of the lectins used above was applied to LNCaP. The fucose-binding probe, modified AAL, yielded over 70 unique glycoproteins. The WGA yielded similar numbers reflecting the high level of fucosylation in LNCaP. Increasing the amount of fucosylation on the cell surface in LNCaP, relative to PNT2, increased the number of oxidized glycoproteins by a factor of two. There was similarly a large overlap of oxidized glycoproteins between AAL and WGA. The SNA probe oxidized only 22 glycoproteins due to the lower sialylation of LNCaP (Fig. S13†).

Inspection of glycoproteins was similarly consistent with the specificity of the lectins (Fig. S14a†). For example, glycoproteomic analysis of TFR1 (transferrin receptor protein 1) yielded primarily fucosylated glycans at N251. Both AAL and WGA produced oxidation of TFR1 in close proximity to the glycosylation site at M283. However, SNA did not produce oxidized peptides for TFR1. The sensitivity of the lectin was also determined for the cell line. The same observations were noticed by investigating the sensitivity of the lectins in the LNCaP cell line, in which all three lectins yielded more than 60% sensitivity towards the putative targeted proteins (Fig. S14b†).

A comparison between LNCaP (a prostate cancer cell line) and PNT2 (a nonmalignant prostate cell line) was useful for comparing relationships in the protein network. For example, a nonglycosylated protein ACSL1 (long-chain-fatty-acid-CoA ligase 1) and a glycoprotein PPT1 (palmitoyl-protein thioesterase 1) were oxidized by the WGA lectin in LNCaP, however neither was observed in PNT2. Indeed, it has been reported that both ACSL1 and PPT1 were upregulated in prostate cancer cells.^43^ STRING analysis showed that ACSL1 does interact with PPT1, and studies have shown that PPT1 glycosylation can affect its ability to form complexes.^44^ These results do point to a correlation between ACSL1 and PPT1 in prostate cancer that was potentially mediated by glycosylation.

## Discussions

Lectin probes that can label protein targets provide the opportunity to understand the relationships between the lectin, the glycan and polypeptide scaffold. Additionally, they elucidate the environment of the glycoprotein targets by providing the proteins that associate with specific glycoproteins on the cell membrane. Probing lectin specificities purely on the glycan provides an incomplete picture of the lectin–glycan interactions.^45^ The polypeptide backbone plays a significant role in defining the conformation of glycans. However, isolating the protein masks the effects of cellular conditions such as interaction with other molecules/ions due to specific localization.^46^ Indeed, the protein backbone is known to restrict glycan conformation and subcellular localization.^47^ Although lectins are typically used to characterize glycans on cell membranes, this study adds to the very limited research performed *in vitro* to examine the lectin specificity.

The fraction of the putative glycoprotein targets that were oxidatively labeled yields the sensitivity of the lectins. When all lectins were used, the total number of glycoproteins oxidized by the lectin probes corresponded to approximately 70% of all glycoproteins detected with both PNT2 and LNCaP. Although there was a broad diversity in the lectins used in this study, not all glycoproteins were oxidatively labeled by the lectin probes. Unmarked glycoproteins, in general, resulted from at least two reasons, namely the expression levels of specific glycans on glycoproteins were low or there were static and dynamic variations in glycoprotein structures.

The results further demonstrated that specific structural motifs such as linkages can be determined at the glycoprotein level by the Lectin PROXL. SNA and MAL are both sialic acid binding lectins with specificities for α(2,6) and α(2,3) sialylated glycans, respectively. More oxidized proteins were obtained with the SNA probe, indicating a higher expression of α(2,6) sialic acid in the PNT2 cell line. Conversely for fucosylated glycans, PSA with a specificity for core α(1,6) fucose yielded less labeled glycoproteins than AAL with broader specificity. Most fucosylated glycans are generally core fucosylated first, followed by antenna fucosylation. That PSA yielded much less oxidation was due more to the shielding of the core fucose by the polypeptide. The poorest specificities were found for the mannose-binding lectin HHL. In contrast, PHA-E and PHA-L which recognize galactose residues on *N*-glycans had higher specificities and oxidized over 70% of galactose-containing glycoproteins. WGA with broad specificity for *N*-glycans also labeled a larger fraction of the glycoproteins. Nonetheless, it too had a unique, previously reported specificity as it appeared to favor hybrid-type over complex-type glycans.

The Lectin PROXL method also revealed cell surface networks that were mediated by specific glycosylation. The nonglycosylated proteins oxidized by the probes were more consistent with glycan-binding proteins that were oxidized due to their proximity to the glycoproteins. By constructing the interaction networks associated with the lectins, we noticed several glycoproteins that behaved as hubs by simultaneously interacting with several other proteins. For example, EGFR was found to interact with many other glycoproteins and nonglycosylated proteins. Indeed, EGFR plays a central role in many biological processes and is associated with many diseases.^48^ Thus, along with EGFR, the nonglycosylated protein catenins such as CTNA1 (catenin alpha-1), CTNB1 (catenin beta-1), and CTND1 (catenin delta-1) were oxidized by the SNA and AAL probes. The interactions between EGFR and catenins have specifically been shown to rely on the glycosylation of EGFR.^49–51^ Other known interactions of glycoproteins interacting with other glycoproteins were also obtained in these interaction maps. For example, EGFR and the glycoprotein ITGB4 (integrin beta-4) were both oxidized by SNA. Here too, *N*-glycans on EGFR were reported to mediate the association between the two glycoproteins.^52^ Other associating proteins were also found to be potentially mediated by glycans. For example, the glycoprotein ENPL (endoplasmin) was oxidized by HHL, while CAV1 (Caveolin-1), a nonglycosylated protein, was also oxidized. Examination of the HHL proteins by STRING predicted that both CAV1 and ENPL are strongly interacting proteins. Comparison of the proteins marked by HHL with other lectins, for example SNA and AAL, did not yield the same glycoprotein-protein interaction map, suggesting that the interaction map may be mediated by high mannose glycosylation, rather than either sialylated or fucosylated glycans. This result therefore suggested that the interactions between glycoprotein and nonglycosylated proteins depended on the glycan structures, perhaps as expected, but now more specifically elucidated. High mannose glycosylation on the cell membrane is important and has been found to play a role in the migration and invasion of the cells by strengthening extracellular protein complexes.^53^

Lectin PROXL is a new addition to the glycobiology toolbox that reveals the blind spot that limits traditional lectin-based analysis. It identifies the protein scaffold of the glycans as well as the associating proteins in the complex. More specifically, it also yields glycan composition and the site-specific localization. As aberrant glycosylation is a hallmark of many diseases including cancer, it will be valuable in developing new targets and new therapeutics. Moreover, the method is not limited to lectins. Future publications will undoubtedly widen the utility of Lectin PROXL to include antibodies and other proteins whose targets on tissues and cell membranes are highly desirable.

## Methods

### Materials

PNT2 and LNCaP cell lines were obtained from the American Type Culture Collection (ATCC). Lectins were purchased from Vector Laboratories. FeBABE was purchased from Dojindo Molecular Technologies. Dithiothreitol (DTT), iodoacetamide (IAA), DBCO-NH_2_, and DBCO-Cy3 were purchased from Sigma-Aldrich. Phosphate Buffered Saline (PBS), Roswell Park Memorial Institute (RPMI) 1640 medium, fetal bovine serum (FBS), penicillin, NHS-PEG4-Azide, and Hochest 33342 were purchased from ThermoFisher Scientific. Sequencing Grade Modified Trypsin was purchased from Promega.

### Modification of lectin with the Fe(iii) probe

The lectin was dissolved in PBS at a final concentration of 1 mg mL^−1^, followed by adding 10 μL of NHS-PEG4-Azide (100 mM) to the lectin solution. The reaction was carried out at room temperature for 1 hour, followed by adding 10 μL of Tris buffer (1 M) to quench the reaction and the excess reagent was removed using the ultra-centrifugal filter. DBCO-FeBABE was prepared in advance, and detailed procedures for synthesizing DBCO-FeBABE were described previously.^24^ 10 μL of DBCO-FeBABE (100 mM) was added to the mixture, and the reaction was carried out in a water bath at 37 °C for 4 hours. The oxidation probe-modified lectin was further purified using the 10k ultra-centrifugal filter.

### Cell culture

Human immortalized normal prostatic epithelial PNT2 cells and human prostate carcinoma epithelial cells LNCaP were obtained from ATCC and grown in Roswell Park Memorial Institute (RPMI) 1640 medium supplemented with 10% (v/v) fetal bovine serum (FBS) and 1% (v/v) penicillin. The cells were maintained in a humidified incubator at 37 °C with 5% CO_2_ and subcultured at 80% confluency.

### Confocal imaging of lectin

Human immortalized normal prostate epithelial PNT2 cells were obtained from ATCC and cultured in FluoroDish™ cell culture dishes (WPI, FL). At around 60% confluency, the cells were fixed with 4% paraformaldehyde. 5 μg of fluorescein-conjugated lectin or DBCO-Cy3-conjugated lectin was added to the cells, and the labeling of cell surface glycans was performed at 37 °C for 30 minutes, followed by staining the nucleus with Hoechst 33342 at 37 °C for 10 minutes. Confocal images were captured using a Leica TCS SP8 STED 3X Super-Resolution Confocal Microscope (Wetzlar, Germany). The images were analyzed and processed using Imaris software (Oxford Instruments, Switzerland).

### Oxidation of cell surface glycoproteins

For oxidative mapping of the sialic-acid environment on the cell surface, 3 × 10^6^ cells were treated with serum-free media supplemented with 20 μg of the lectins modified with the oxidation probe for 30 minutes at 37 °C. The cells were washed with PBS three times to wash off unbound lectins, followed by the treatment with 100 μM hydrogen peroxide and sodium ascorbate for 30 minutes at 37 °C. The hydroxyl radicals were quenched with 10 mL of 10 mM methionine amide hydrochloride in PBS. Cells were harvested and resuspended in a homogenization buffer containing protease inhibitor cocktail (EMD Millipore, CA), 0.25 M sucrose, and 20 mM HEPES-KOH (pH 7.4). Cells were lysed at 4 °C using a probe sonicator (Qsonica, CT) performed with alternating on and off pulses of 5 and 10 seconds, respectively.

### Cell membrane extraction

Detailed procedures for cell membrane extraction were described previously.^47^ Briefly, the nucleus and cellular debris were isolated by centrifugation at 2000×*g* for 10 minutes. The mitochondrial fraction was removed by centrifugation at 12 000×*g* for 10 minutes. The supernatants were subjected to ultra-centrifugation at 200 000×*g* for 45 minutes to extract the membrane fraction. The crude membrane fraction was further washed with 500 μL of Na_2_CO_3_ (0.2 M) and nanopure water, respectively.

### Protein digestion and purification

The cell membrane pellets were reconstituted with 60 μL of 8 M urea and sonicated for 15 minutes for denaturing. 2 μL of DTT (550 mM) was added to the samples and incubated for 50 minutes at 55 °C, and the free cystine was alkylated with 4 μL of IAA (450 mM) for 20 minutes at room temperature in the dark. 30 μL of trypsin (0.1 μg μL^−1^) was added to the mixture and the PH of the solution was adjusted using 420 μL of ammonium bicarbonate (3.95 mg mL^−1^). The tryptic digestion was performed at 37C for 18 hours. The resulting peptides were purified using solid-phase extraction with C18 cartridges. The trypsin digestion of glycoproteomics samples was prepared the same as proteomic analysis, and iSPE®-HILIC cartridges (The Nest Group, MA) were used to enrich the glycopeptides.

### Proteomic analysis using LC-MS/MS

The proteomics and glycoproteomics samples were characterized using an UltiMate™ WPS-3000RS nanoLC system coupled with an Orbitrap Fusion Lumos (ThermoFisher Scientific). 1 μL of the sample was injected, and the analytes were separated on an Acclaim™ PepMap™ 100C18 LC Column (3 μm, 0.075 mm × 250 mm, ThermoFisher Scientific) at a flow rate of 300 nL min^−1^. Water containing 0.1% formic acid and 80% acetonitrile containing 0.1% formic acid were used as solvents A and B, respectively. MS spectra were collected with a mass range of *m*/*z* 600–2000 at a rate of 1.5 s per spectrum in positive ionization mode. The filtered precursor ions in each MS spectrum were subjected to fragmentation through 30% higher-energy C-trap dissociation (HCD) with nitrogen gas.

### Data analysis

Oxidized proteins and glycoproteins were identified using Byonic software (Protein Metrics, CA) against the human protein database (UniProt). Alkylation of cysteine with carbamidomethylation was assigned as a fixed modification. Deamidation of asparagine and glutamine, methylation of lysine and arginine, and acetylation of the protein N-terminus were assigned as the rare variable modifications. For oxidized samples, the oxidized modifications were selected as common variable modifications according to previous settings. For glycoprotein samples, an in-house human *N*-glycan database was applied for asparagine *N*-glycosylation. The unmodified and modified peptides were then quantified using Byologic. The extent of modification was calculated by dividing the abundance of the modified peptides to the total abundance of corresponding wildtype and modified peptides.

### Cell surface *N*-glycomic analysis

The cell membrane fractions were resuspended with 100 μL of 5 mM DTT in 100 mM ammonium bicarbonate. The mixture was heated in boiling water for 3 minutes. The cleavage of *N*-glycans was performed by adding 2 μL of PNGase F followed by incubation in a 37 °C water bath overnight. The released *N*-glycans were separated using centrifugation at 200 000×*g* for 30 minutes, and the supernatant was purified using porous graphitic carbon (PGC) on an SPE plate. The glycan samples were dried and reconstituted in 30 μL nanopure water. 5 μL of the sample was injected and analyzed with an Agilent 6520 Accurate Mass Q-TOF LC/MS equipped with a PGC nano-chip (Agilent, CA). A binary gradient using solvent A with 3% (v/v) ACN and 0.1% (v/v) formic acid in water and solvent B with 90% (v/v) ACN and 1% (v/v) formic acid in water was applied to separate *N*-glycans at a flow rate of 300 nL min^−1^. The resulting chromatographs of glycans were extracted with the MassHunter Qualitative Analysis B08 software (Agilent, CA). *N*-glycan compounds were identified with an in-house library that contains the accurate mass and formula of human *N*-glycans, and the *N*-glycan structures were confirmed through tandem MS fragmentation.

## Author contributions

Y. X. designed and performed experiments, analyzed data, created the figure and wrote the manuscript. Y. S. performed experiments and analyzed data. Q. L, S. J., and J. R, performed experiments. C. B. L conceived the idea, supervised the study, and co-wrote the manuscript.

## Conflicts of interest

There are no conflicts with this report.

## Supplementary Material

SC-011-D0SC04199H-s001

SC-011-D0SC04199H-s002

SC-011-D0SC04199H-s003

SC-011-D0SC04199H-s004

SC-011-D0SC04199H-s005

SC-011-D0SC04199H-s006

SC-011-D0SC04199H-s007

SC-011-D0SC04199H-s008
