# Supplementary material for: Determination of the glycoprotein specificity of lectins on cell membranes through oxidative proteomics
Source: Chem Sci. 2020 Aug 17;11(35):9501–12. doi: 10.1039/d0sc04199h (PMC8162070; doi:10.1039/d0sc04199h)
Supplement: SC-011-D0SC04199H-s001 [file SC-011-D0SC04199H-s001.pdf]

Electronic Supplementary Material (ESI) for Chemical Science.
This journal is © The Royal Society of Chemistry 2020

Determination of the glycoprotein specificity of lectins on cell membrane through oxidative proteomics

Supplementary Information

Yixuan Xie[1]; Ying Sheng[2]; Qiongyu Li[1]; Seunghye Ju[1]; Joe Reyes[4]; Carlito B Lebrilla[1,3]

[1]Department of Chemistry, University of California, Davis, Davis, California, United States. [2]Department of Chemistry, Biochemistry, Molecular, Cellular and Developmental Biology Graduate Group, University of California, Davis, Davis, California, United States. [3]Department of Biochemistry, University of California, Davis, Davis, California, United States. [4]Marine Science Institute, University of the Philippines, Diliman, Quezon City, Philippines. Correspondence and requests for materials should be addressed to C.B.L. (email: cblebrilla@ucdavis.edu)

Supplementary Data 1. Summary of oxidized glycoproteins on PNT2 cells.

Supplementary Data 2. Summary of oxidized nonglycosylated proteins on PNT2 cells.

Supplementary Data 3. Glycoproteomic results of PNT2 cell line.

Supplementary Data 4. Glycoproteomic results of α(2,3)sialidase-treated PNT2 cell line.

Supplementary Data 5. Summary of oxidized glycoproteins on LNCaP cells.

Supplementary Data 6. Summary of oxidized nonglycosylated proteins on LNCaP cells.

Supplementary Data 7. Glycoproteomic results of LNCaP cell line.

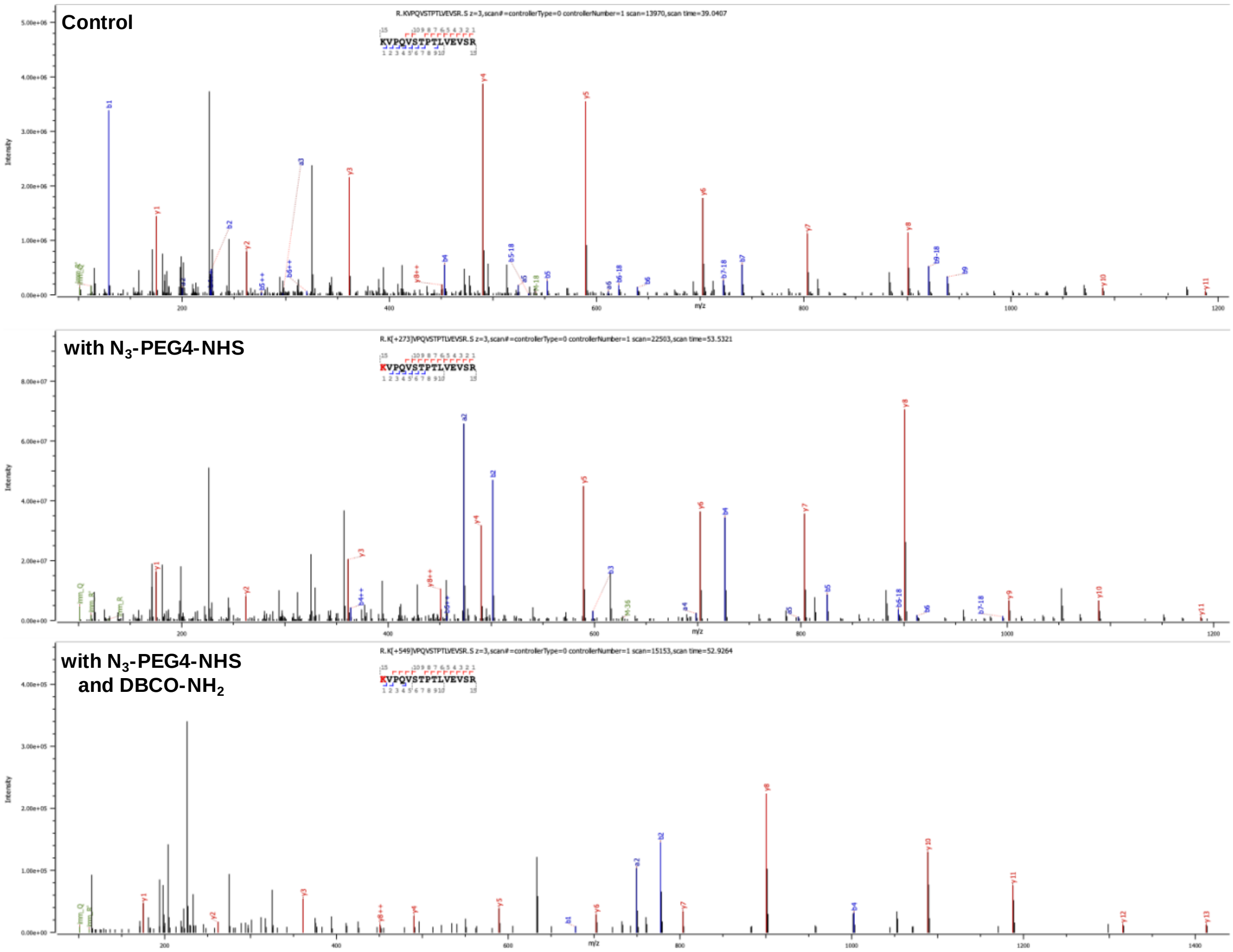

Figure S1. The extent of reaction was determined using a model protein, BSA. The product peptides were characterized by nanoLC-MS. The tandem MS/MS data showed the modification with azido group (+273.13 Da) and DBCO (+549.26 Da) at K437 residue.

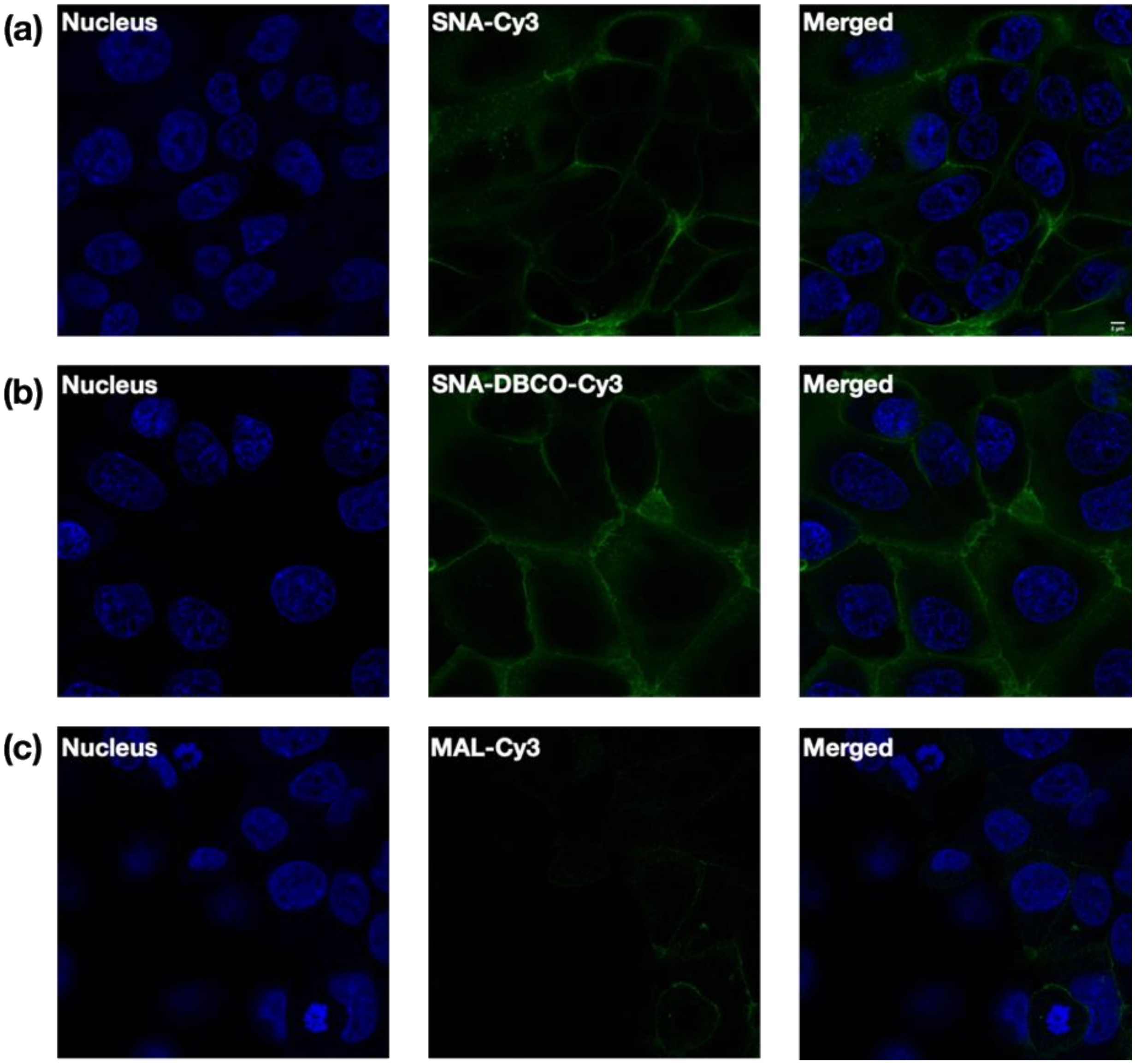

Figure S2. Validation of the binding efficiencies of the modified lectins using confocal microscopy. PNT2 Cells were treated with **(a)** SNA-Cyanine3 (control), **(b)** SNA-DBCO-Cyanine3 (modified), and **(c)** MAL-Cyanine3. The cell nucleus was stained with Hoechst 33342.

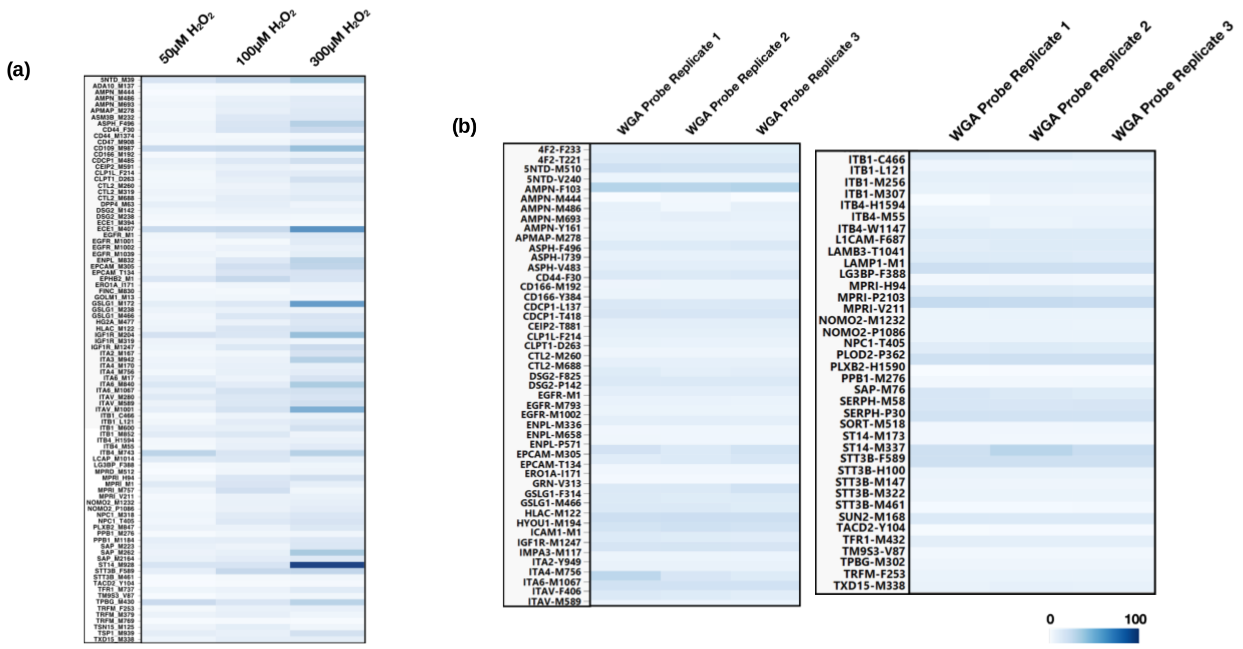

Figure S3. (a) Optimization of the conditions for the labeling reaction. The extent of oxidation quantified using Byologic software. Each column represents a single treatment condition, and each row represents the oxidation sites of selected proteins. **(b)** Quantification results from three consecutive LC-MS injections of WGA probe-oxidized proteins.

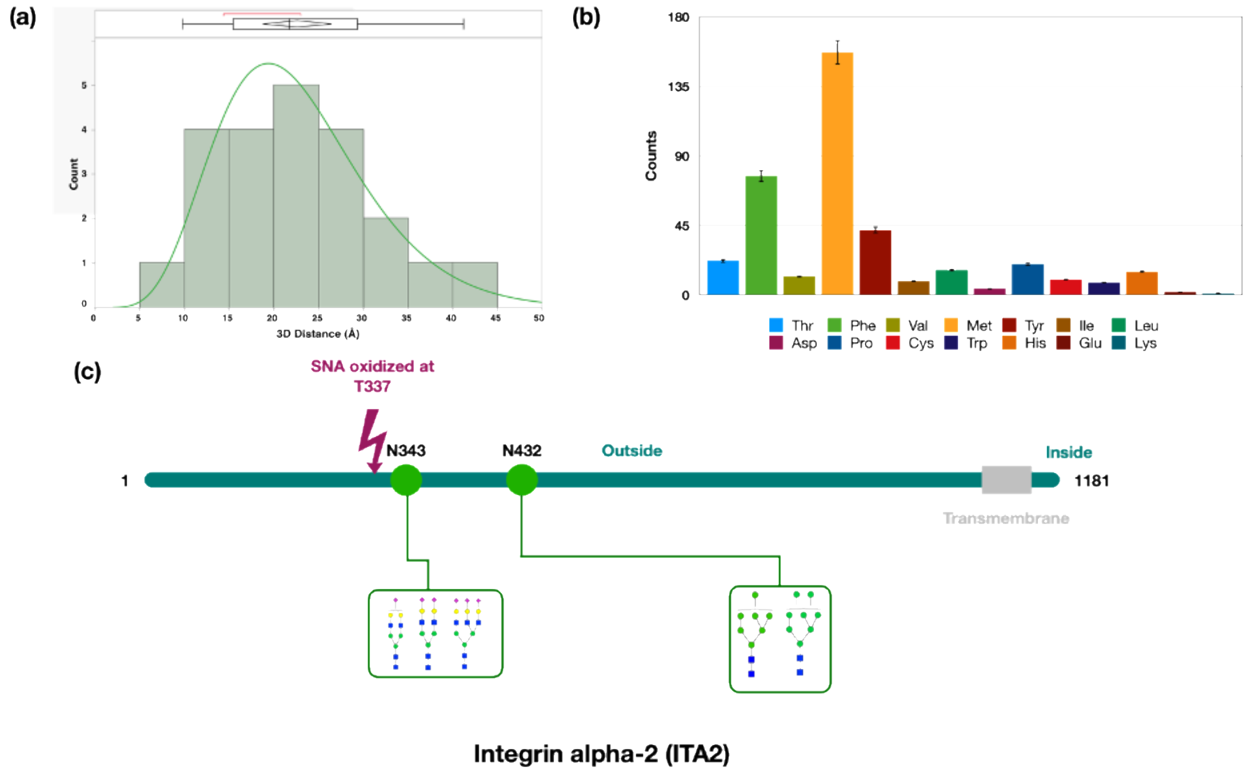

Figure S4. (a) The distribution of distances in oxidized glycoproteins between the site of oxidation and glycosylation for WGA. **(b)** Frequency of oxidized amino acid residues observed from all eight lectin probes. **(c)** The relationship between glycosylation sites and the site of oxidation on the glycoprotein ITA2 (integrin alpha-2).

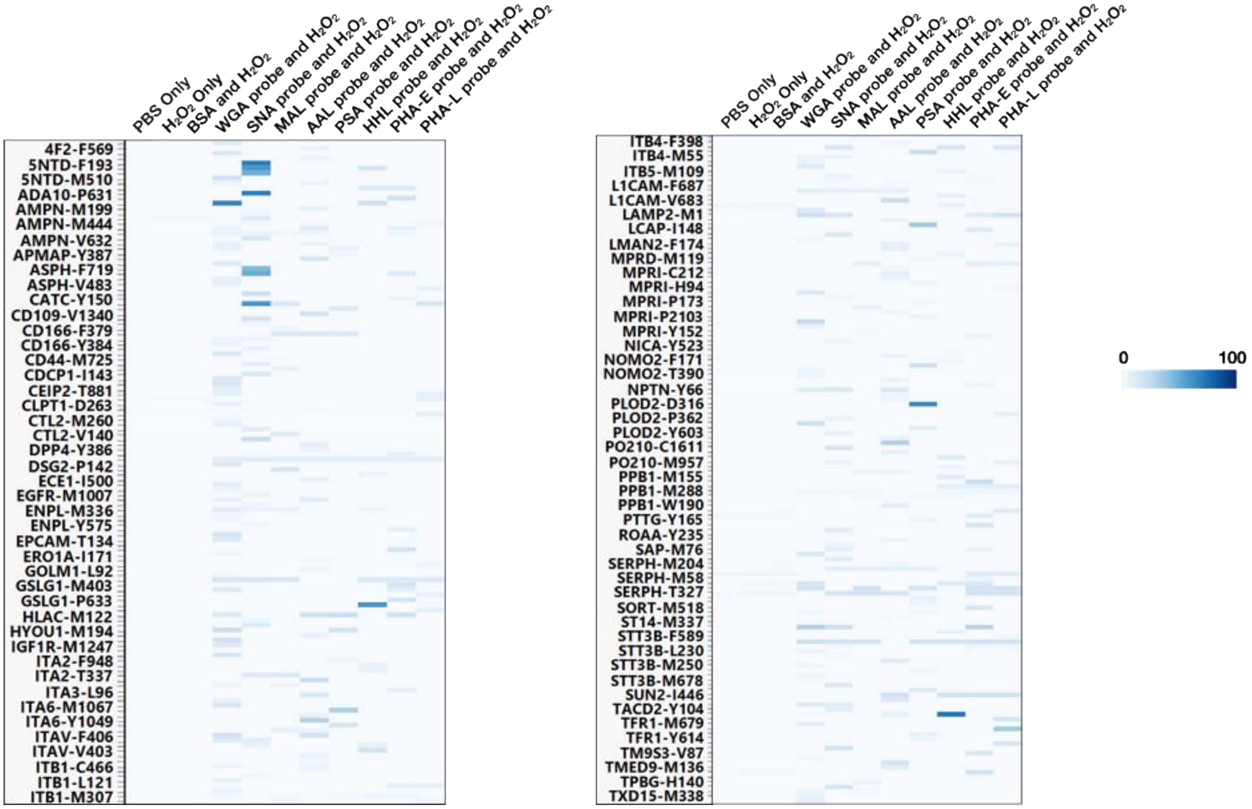

Figure S5. The extent of oxidation quantified by Byologic for glycoproteins oxidized by the lectin probes.

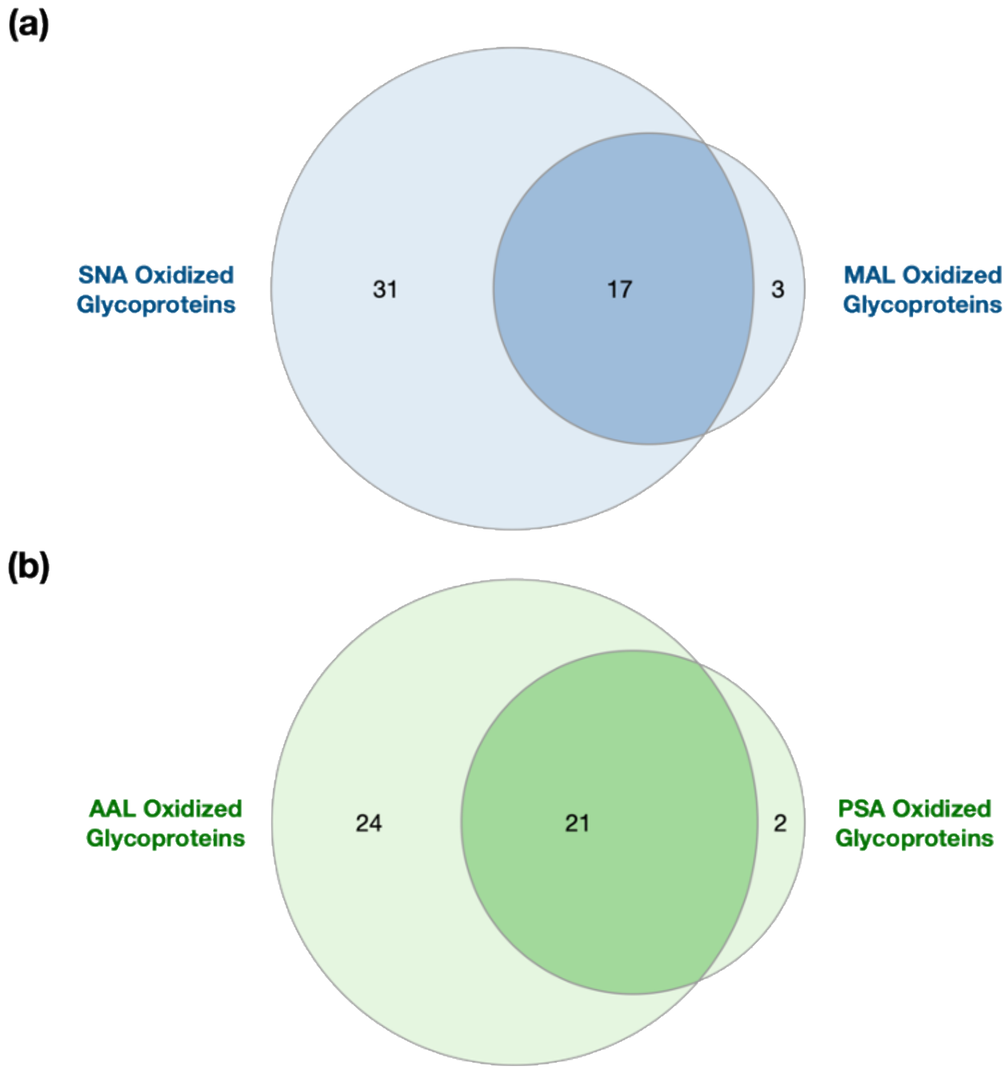

Figure S6. The overlap in oxidized glycoproteins between different lectins. **(a)** Glycoproteins were found oxidized by both sialylated glycan-binding lectins, SNA and MAL. **(b)** Glycoproteins were found oxidized by both fucosylated glycan-binding lectins, AAL and PSA.

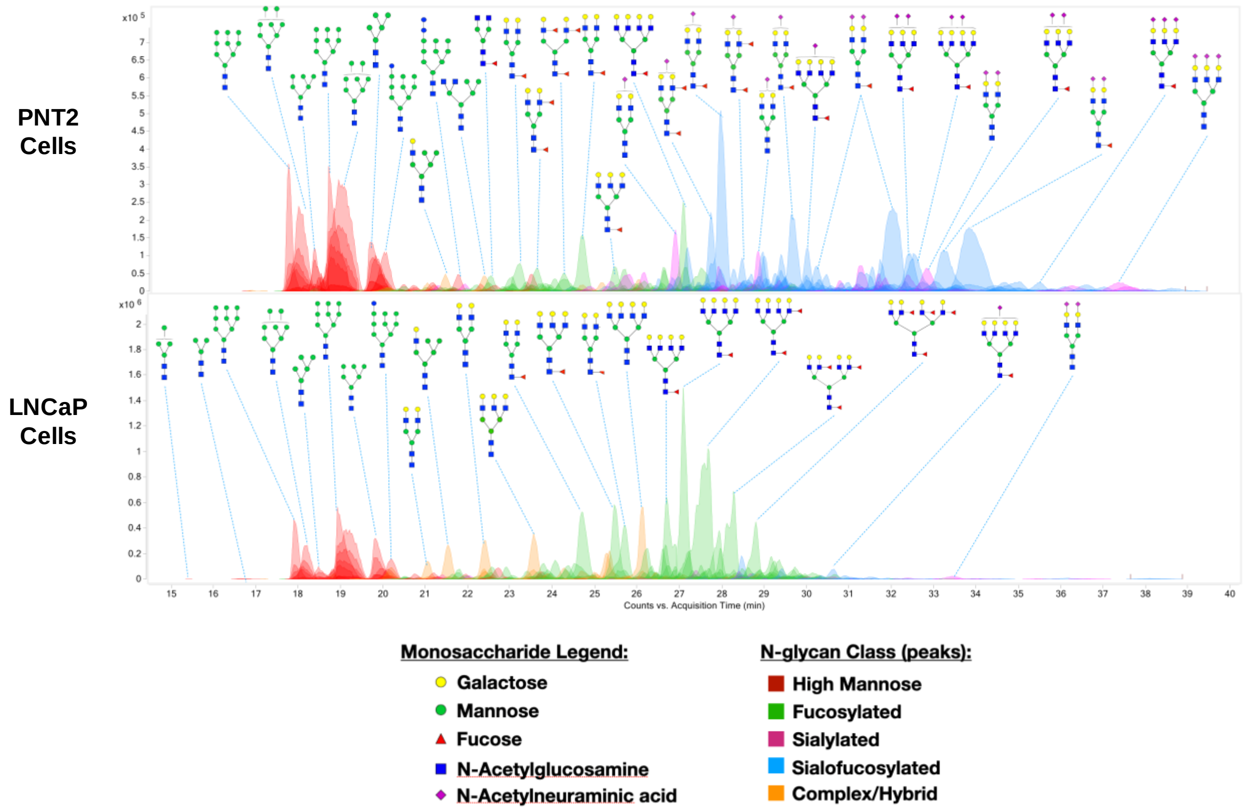

Figure S7. LC-MS profile of N-Glycans released from PNT2 cells (top) and LNCaP cells (bottom). Annotated structures are putative based on mass and compositions. LC-MS peaks were color coded to assign glycan subtype.

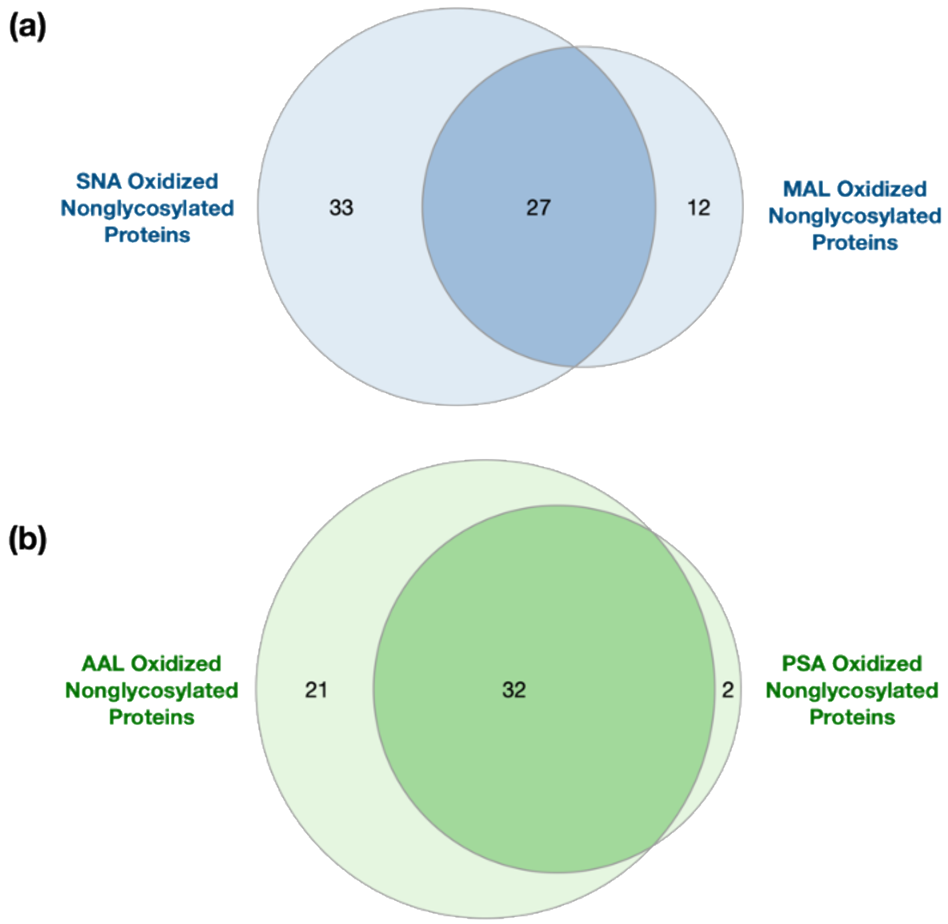

Figure S8. The overlap of oxidized nonglycosylated proteins between different lectins.

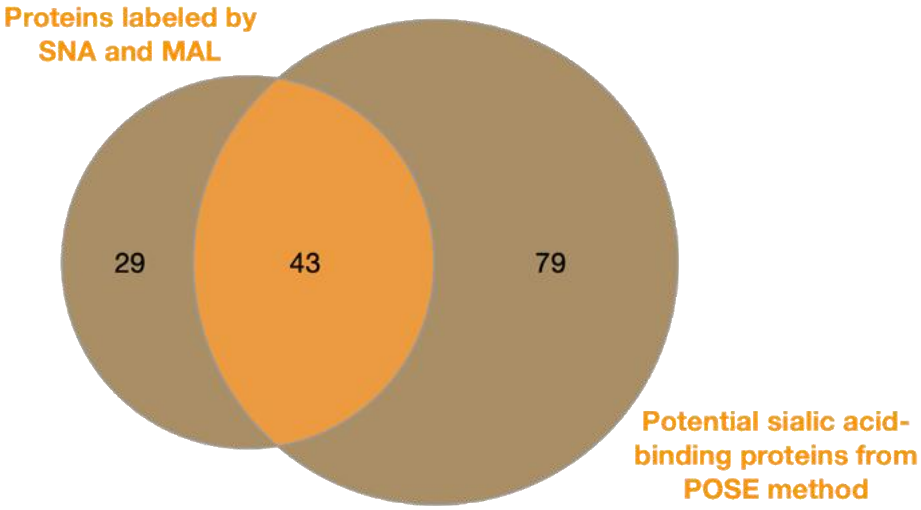

Figure S9. Proteins labeled by the sialylated glycan-binding lectins were found to overlap with the potential sialic acid-associated proteins as determined by a previously used method POSE to determine sialic acid binding proteins.

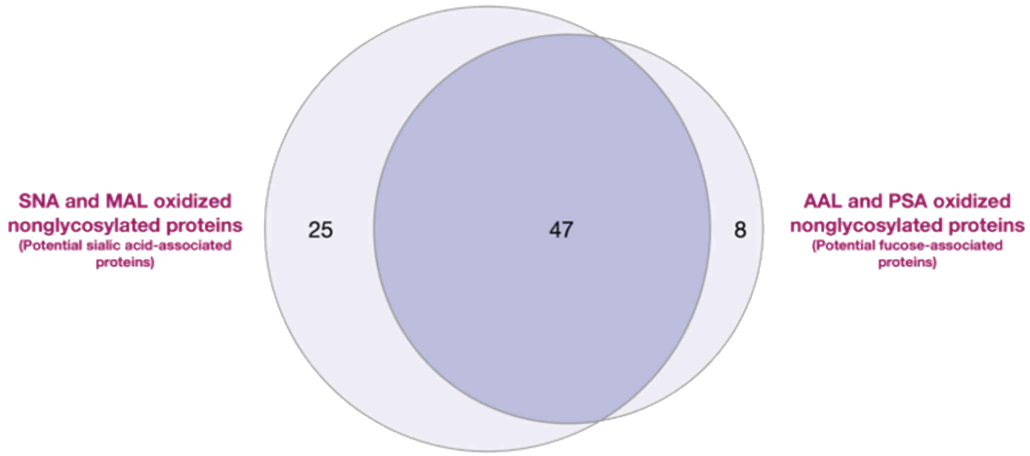

Figure S10. Nonglycosylated proteins oxidized by specific lectins. These proteins were oxidized due to their proximity to the respective glycosylated protein. The large overlap supports the observation that show most of the glycans on the cell membrane are both sialylated and fucosylated.

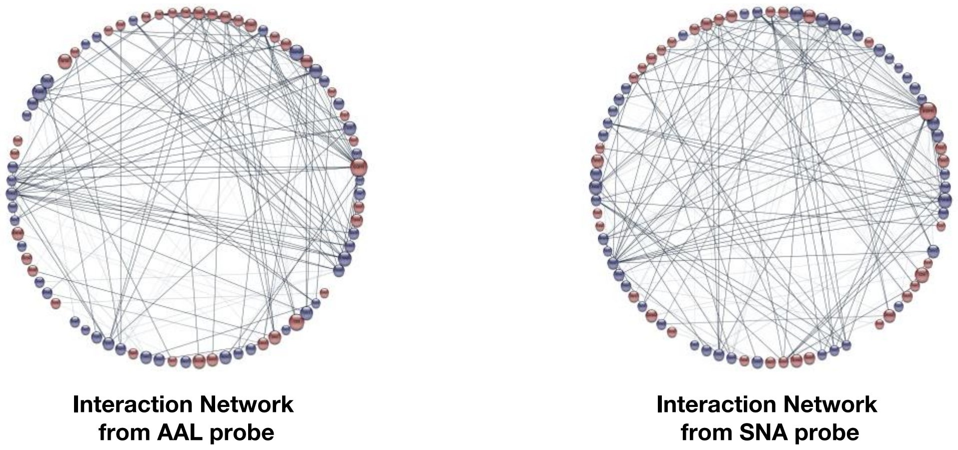

Figure S11. The interaction networks probed by AAL lectin (left) and SNA lectin (right).

(a)

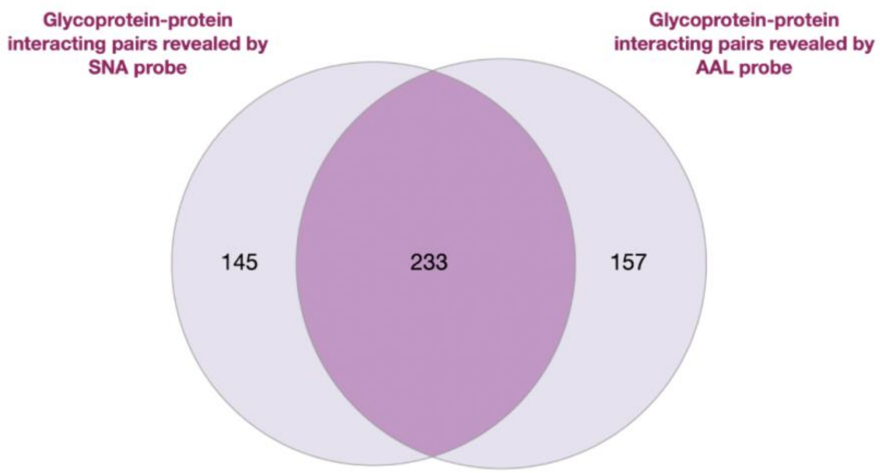

(b)

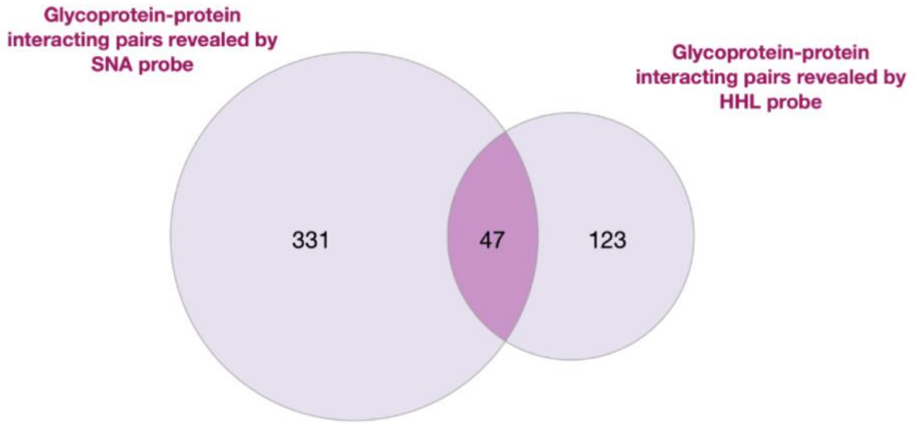

Figure S12. (a) The overlapping interaction network found common between SNA and AAL probes. **(b)** The interaction network found common between SNA and HHL probes.

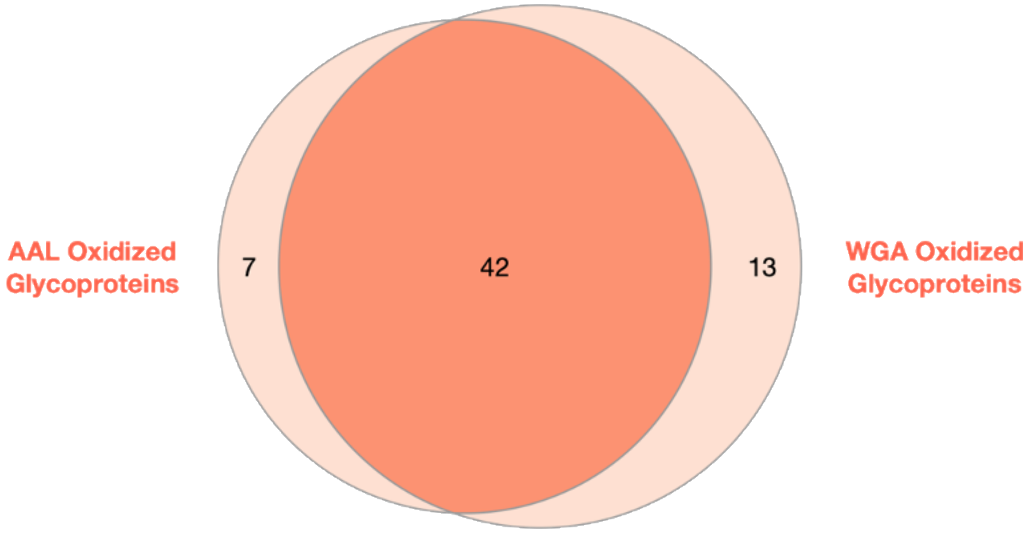

Figure S13. The oxidized glycoproteins overlap between lectin WGA and lectin AAL from LNCaP cell line.

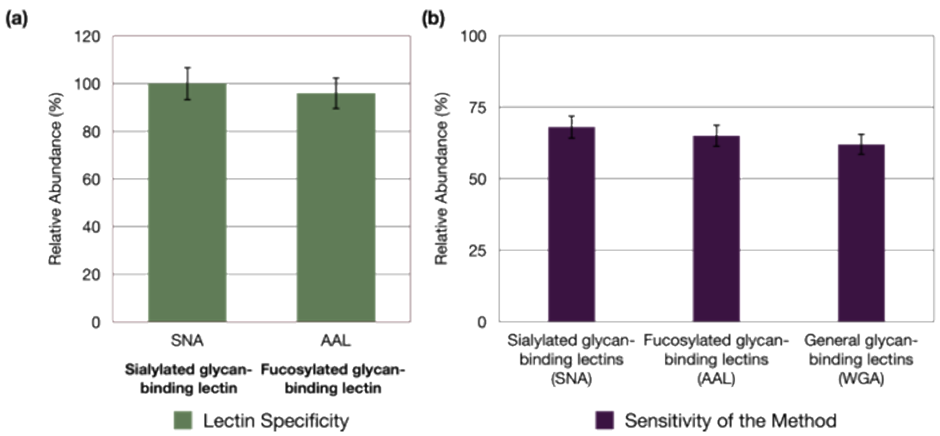

Figure S14. (a) The glycoprotein specificity of lectins on LNCaP cells. **(b)** The sensitivity of the method on LNCaP cells. The error bars were obtained based on triplicate results.
